# Cold Philosophy

**Published:** 1860-11

**Authors:** 


### COLD PHILOSOPHY.

WHEN Patrick was asked what he would take to climb a steeple one frosty morning, "I'll take a cold, yer honor, be sure," was the ready reply. Sandy, standing hard-by, said he would "take a dollar." It may be practically useful to know how a cold acts on the system. Colds always come from outside agencies. In health, from two to six pounds of waste and impure matter, in the shape of fluid and gas, is passed from the interior body towards the surface; the skin is perforated by millions of little holes, through which this waste is poured outside the body; a good deal of it dries and forms into flakes. In health, these holes or "pores" are open, known by a "soft feel" of the skin; they are kept open by warmth, but close instantly on the application of cold; if the closure has been sudden, decided, or general, a feeling is caused, familiarly known as a "chill;" these waste and impure fluids, not being able to have an exit through their natural channels, retreat and seek a place of escape elsewhere; if they find it instantly, as in an attack of loose bowels, the shock to the system is expended in that direction, and the cold is cut short off; the same if the person is seized with an attack of vomiting, or of violent bleeding at the nose, or an excessive watering at the nose, or of an accidental wound causing the loss of a large quantity of blood. It is as if the natural vent of a steam-engine were closed while in operation: if an equal "vent" is made in another direction, all is well; and the vent must be had, or an explosion is inevitable. But before this vent is made, in case of a cold having been taken, and the arrested outgoing fluids not having as yet found egress, there is that much more of actual matter in the system than it is accustomed to, making us feel "stuffed up," "full," "oppressed." Most expressive and literally true are these phrases, and until a vent is made, the fuller and fuller does the body become. We express ourselves as feeling "bad all over," and no wonder, for every blood-vessel in the body is not only fuller than it ought to be, but it is filled with a fluid made up of the pure blood, mixed with all the impurities which would otherwise have been thrown out of the system as effete matter; and the blood of the whole body being impure, imperfect, feeling, taste, appetite, every bodily sense is deranged, the mind participates in the general disorder, and petulance and ill-nature pervade the whole deportment, and what the sufferer feels, others see, that he is "as cross as a bear."

If, however, within a few hours after a felt chill, or after a cold has been taken, and before the current has become in a measure fixed in its unnatural direction inwards, the "pores" of the skin are reöpened, that current is turned back and harm is avoided; hence the efficacy of what is called the "old woman's remedy," "a good sweat," produced by putting the patient to bed, "tucking in" the bed-clothes, and pouring down a gallon, more or less, of hot "catnip-tea," or any other hot drink. We have pleasant memories of the good taste of a "stew," a mixture of Bourbon whisky, hot water, sugar, a little butter, and hot spices. Oh! how good it was! It's a medicine we always take with pleasure, but we don't advise others to do so—it's dangerous, very! its ultimate effects have been the death of many a noble-hearted fellow.

But if all the discomfort of a cold is caused by an unusual amount of matter being shut up in the system, is it not the most consummate folly to eat an atom of any thing, or drink a drop of water, to increase the "fullness" of the body? We should instead, the very moment a chill has been experienced, or that we in any other way become sensible of the fact that we have taken cold, set about doing two things: first, get up a feeling of warmth in the body, even if it requires a room to be heated to two hundred degrees of Fahrenheit, and keep it at that point until perspiration has been induced, and continued for some hours; in addition, do not eat an atom of food, at least until next day, or until you are conscious that the cold has been broken; and then, for a few days, live exclusively on soups, crust of cold bread, hot teas, and fruits.

Let it be kept in remembrance that every mouthful of food, even of the mildest, a man swallows from the instant the cold has been taken, only makes a proportional amount of phlegm to be coughed up. "Feed a cold and starve a fever," is a tremendous lie. Starve them to death, as we would a garrison, by cutting off supplies, and the fortress will be yielded within thirty-six hours, if the process be begun within twelve hours after the cold has been taken. If a chill has been experienced, begin on the instant to stop supplies, and then to cause an artificial drain, by the means already named for inducing free perspiration; in this manner, the very worst colds will be arrested, will be cut short off in four cases out of five. Unfortunately, the first effect of a cold is to increase the appetite, the indulgence of which protracts the cold to days and weeks, with this result, that after the first two or three days, food becomes an aversion, and there is no appetite for weeks together sometimes; better, then, starve willingly for a day or two than be unable to eat any thing for a fortnight, to say nothing of the troublesome coughing and other discomforts during the whole of that time.

